# First Isolates of *Leptospira* spp., from Rodents Captured in Angola

**DOI:** 10.4269/ajtmh.15-0027

**Published:** 2016-05-04

**Authors:** Elsa Fortes-Gabriel, Teresa Carreira, Maria Luísa Vieira

**Affiliations:** Grupo de Leptospirose e Borreliose de Lyme, Unidade de Microbiologia Médica, Global Health and Tropical Medicine (GHTM), Instituto de Higiene e Medicina Tropical (IHMT), Universidade Nova de Lisboa (UNL), Lisbon, Portugal

## Abstract

Rodents play an important role in the transmission of pathogenic *Leptospira* spp. However, in Angola, neither the natural reservoirs of these spirochetes nor leptospirosis diagnosis has been considered. Regarding this gap, we captured rodents in Luanda and Huambo provinces to identify circulating *Leptospira* spp. Rodent kidney tissue was cultured and DNA amplified and sequenced. Culture isolates were evaluated for pathogenic status and typing with rabbit antisera; polymerase chain reaction (PCR) and sequencing were also performed. A total of 37 rodents were captured: *Rattus rattus* (15, 40.5%), *Rattus norvegicus* (9, 24.3%), and *Mus musculus* (13, 35.2%). Leptospiral DNA was amplified in eight (21.6%) kidney samples. From the cultures, we obtained four (10.8%) *Leptospira* isolates belonging to the Icterohaemorrhagiae and Ballum serogroups of *Leptospira interrogans* and *Leptospira borgpetersenii* genospecies, respectively. This study provides information about circulating leptospires spread by rats and mice in Angola.

Leptospirosis is a widespread zoonosis caused by spirochetes of the genus *Leptospira*, with a recognized high incidence in tropical countries.^1^ Rodents are the natural reservoirs of pathogenic leptospires and the main host responsible for its transmission to humans.^2^–^4^ Outbreaks of leptospirosis have been associated with high-level infestation of rodents, floods, and occupational activities. However, in many countries, morbidity and mortality due to leptospirosis are underestimated.^1^–^4^

In Africa, few reports about human leptospirosis are available.^5^ In Angola, a serological survey was conducted by Baptista in 1991 in Huila province among cattle; this study revealed the presence of antibodies against *Leptospira interrogans* sensu lato in 35% of 1,518 animals analyzed (unpublished data). We recently performed a serological and epidemiological survey, in 650 febrile patients in Luanda and Huambo provinces. We found evidence of infection by *Leptospira* serovars belonging to Icterohaemorrhagiae, Pomona, and Ballum serogroups (E. Fortes-Gabriel, 2013, personal communication). Responses to questionnaires revealed that more than 50% of individuals surveyed had seen rodents near their houses. We now report a characterization of rodent *Leptospira* species in the Luanda and Huambo provinces.

We trapped rodents in February and April 2013 during the rainy season in Luanda (8°40′S and 13°40′E) and Huambo (12°45′S and 15°45′E) provinces, respectively (Figure 1

**Figure 1.**
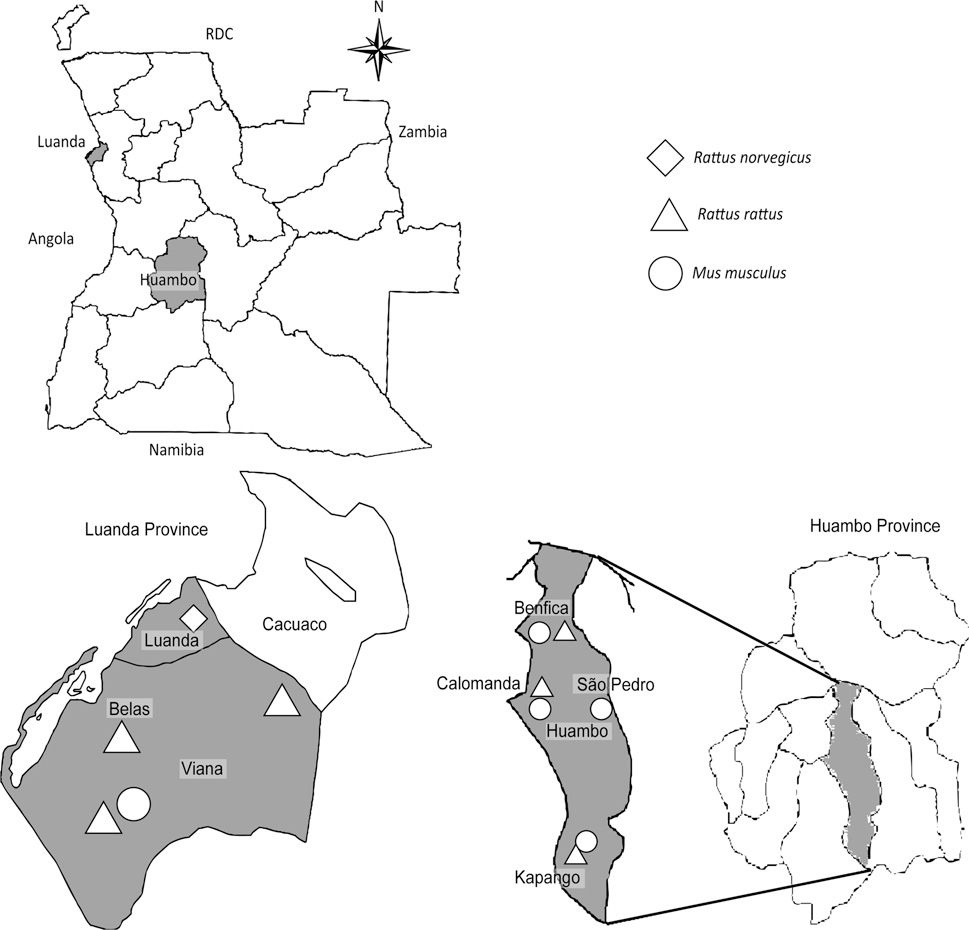
Schematic map showing the location of capture and respective rodent species distribution.

). About 50 handmade live traps (Tomahawk type) were distributed randomly in 20 different urban and rural neighborhoods. Morphometric parameters of rodents were recorded to identify the species. Rodent kidneys were aseptically collected following biosafety and animal welfare guidelines.^6^

For culture, one kidney from each animal was homogenized in 10 mL Ellinghausen, McCullough, Johnson, and Harris (EMJH, Difco, BD Diagnostics, Sparks, MD) liquid medium, and 0.5 mL of the suspension was inoculated in EMJH semisolid medium following incubation at 29°C. The other kidney was frozen at −20°C and stored for molecular assays.

Cultures and kidneys were transported to the Reference Laboratory for Leptospirosis at the Instituto de Higiene e Medicina Tropical, Universidade Nova de Lisboa (UNL). Cultivation was continued for 2 months at 29°C, and culture tubes were assessed every week by dark field microscopy.

Culture isolates were differentiated according to previously described methods,^7^,^8^ using two reference *Leptospira* serovars [Copenhageni (strain M20) and Patoc (strain PatocI)] as pathogenic and saprophytic controls, respectively. Cultures (10^8^ cells/mL) of each isolate were analyzed for: 1) growth at 13°C and 29°C; 2) growth in EMJH with and without 8-azaguanine 225 μg/mL; and 3) morphological modification to spherical forms in the presence of 1M NaCl. A microscopic agglutination test with six rabbit antisera for two of the major *Leptospira* serogroups (Icterohaemorrhagiae and Ballum), was also performed.

Genomic DNA from *Leptospira* isolates and kidney samples was extracted with a kit (Citogene^**®**^, Citomed, Lisbon, Portugal) in accordance with the manufacturer's instructions. Polymerase chain reaction (PCR) was performed targeting the *hap1* gene (also designated *lipL32*), which encodes a hemolysis-associated protein,^9^ and with the iRep1 primer (5′-AGC GGG TAT GAGTCC GC-3′),^10^ to compare DNA fingerprints patterns, with the 19 serogroups represented by 23 pathogenic serovars (*L. interrogans* sensu lato) and one saprophytic (*Leptospira biflexa*). Clustering and alignment of DNA sequences from the isolates was performed with ClustalW2/EMB/EBI (http://www.ebi.ac.uk/Tools/msa/clustalw2/). A phylogenetic tree was constructed with Molecular Evolutionary Genetics Analysis (MEGA) version 6,^11^ using the neighbor-joining method, Jukes-Cantal model, and represents 1000 replicates with confidence greater than 50%.

Our study included 37 rodents collected at eight sites from urban and rural areas near households and garbage dumps (Figure 1) in Luanda and Huambo provinces. The rodents were predominantly adult females and identified as *Rattus rattus* (15, 40.5%), *Rattus norvegicus* (9, 24.3%), and *Mus musculus* (13, 35.2%).

Leptospires were successfully isolated from four (10.8%) rodents. These isolates, LDA02, LDA05, LDA10, and HBO34, were from *R. norvegicus* and *M. musculus* species, captured in Luanda and Huambo provinces, respectively. Pathogenic characteristics were demonstrated by phenotypic tests. All four isolates exhibited an agglutination titer of 1:3200 with rabbit antisera (Table 1).

DNA from the four isolates was amplified, and the nucleotide sequences revealed a similarity of 100% with either *L. interrogans* or *L. borgpetersenii* (Table 1). DNA from the kidney samples was also amplified in eight (21.62%) out of a total (*N* = 37) of the samples.

When compared with 24 reference serovars, iRep1-PCR results for our isolates showed a similarity between fingerprint patterns to either the Icterohaemorrhagiae or Ballum serogroups.

The four rodent isolates were assigned GenBank accession numbers LC006258, LC006259, LC006260, and LC006261. A phylogenetic tree (Figure 2

**Figure 2.**
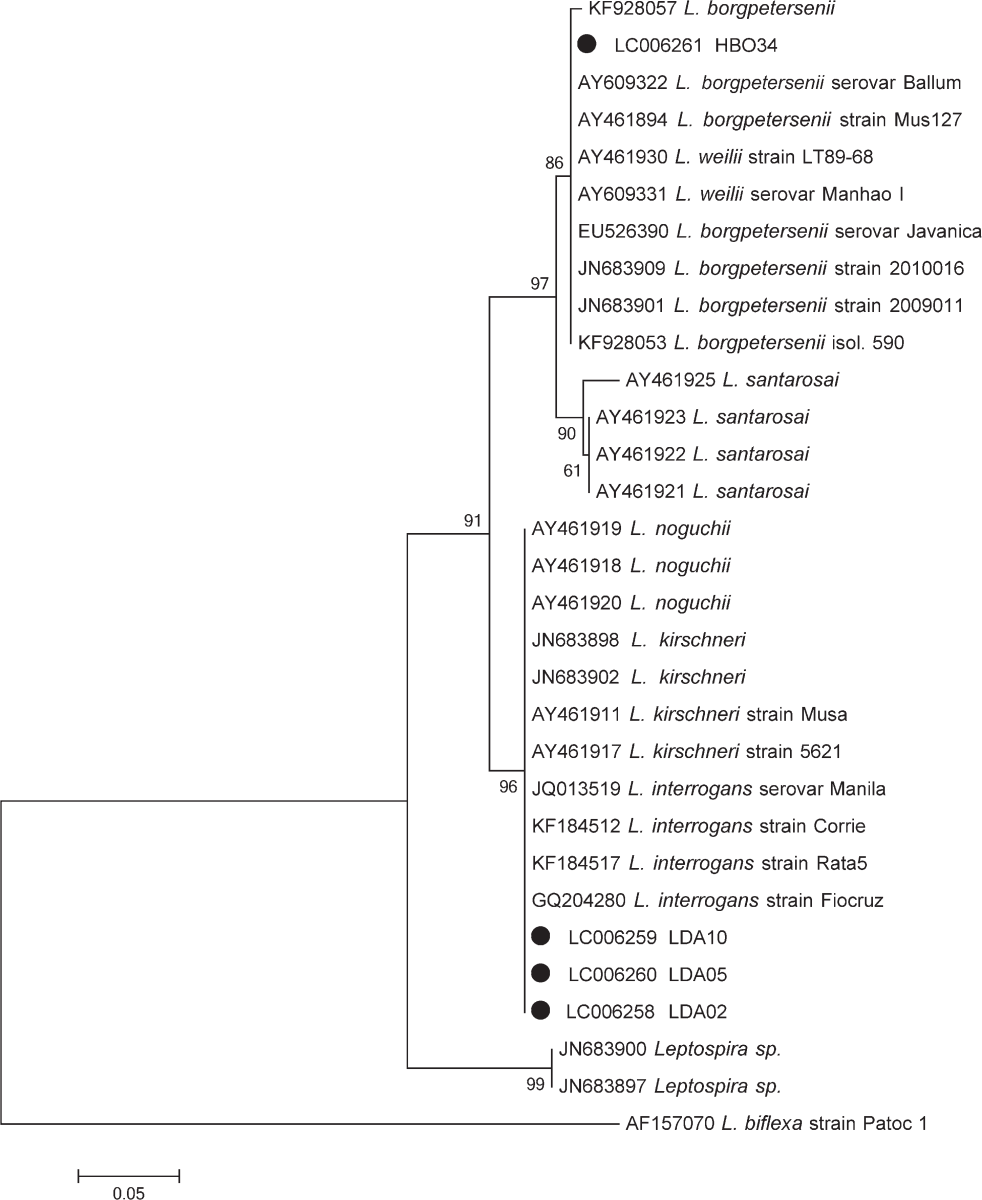
Phylogenetic tree based on partial *hap1* gene sequences from the isolates and the reference strains available in GenBank. Genospecies *Leptospira biflexa* was used as the out-group. Accession numbers of *Leptospira* spp. isolates: LC002658–LC002661.

) demonstrates consensus between the genospecies *L. interrogans* and *L. borgpetersenii* (reference strains and/or homologous genospecies available in GenBank), and the nucleotide sequences from our isolates.

This study offers the first characterization of leptospires in rodents captured in Angola. Our findings confirm data from other countries, suggesting a close relationship between rodents in the environment and the occurrence of human leptospirosis.^12^–^15^ Our field work was carried out during the rainy season, appreciating the importance of seasonality in predicting human infection risk due to an increase in leptospire survival during the rainy season.^12^

Our isolates of *Leptospira* spp., obtained from two of the three most abundant rodent species (*R. norvegicus* and *M. musculus*) in Angola, suggest that these small mammals are a primary infection source of leptospires among the human population. The isolates obtained were classified according to molecular assays as *L. interrogans* and *L. borgpetersenii* genospecies and as belonging to Ballum and Icterohaemorrhagiae serogroups based on rabbit antisera. Results were consistent across all tests (phenotypic, serological, and genotypic).

Phylogenetic analysis revealed six *Leptospira* species partitioned into two clusters showing monophyletic groups. Similar results were describing of the *lipL*32 gene.^16^ Therefore, the present study opens new perspectives on the knowledge of the patterns of *Leptospira* infection among rodents in Angola. Further studies of serovar diversity and rodent reservoirs in this country will be helpful.

## Figures and Tables

**Table 1:** Summary of isolates characterization by phenotypic, serological, and molecular tests

*Leptospira* isolates (rodent species)	Pathogenic status	Rabbit antisera serogroup (reciprocal titer)	iRep-PCR serogroup	PCR-*hap1* genospecies (GenBank accession no.)
LDA02 (*Rattus norvegicus*)	Yes	Icterohaemorrhagiae (1:3200)	Icterohaemorrhagiae	*Leptospira interrogans* (LC006258)
LDA05 (*R. norvegicus)*	Yes	Icterohaemorrhagiae (1:3200)	Icterohaemorrhagiae	*L. interrogans* (LC006259)
LDA10 (*R. norvegicus*)	Yes	Icterohaemorrhagiae (1:3200)	Icterohaemorrhagiae	*L. interrogans* (LC006260)
HBO34 (*Mus musculus*)	Yes	Ballum (1:3200)	Ballum	*L. borgpetersenii* (LC006261)

PCR = polymerase chain reaction.
